# Interaction between *Serotonin Transporter* and *Serotonin Receptor 1 B* genes polymorphisms may be associated with antisocial alcoholism

**DOI:** 10.1186/1744-9081-8-18

**Published:** 2012-05-02

**Authors:** Tzu-Yun Wang, Sheng-Yu Lee, Shiou-Lan Chen, Yun-Hsuan Chang, Shih-Heng Chen, Chun-Hsien Chu, San-Yuan Huang, Nian-Sheng Tzeng, Chen-Lin Wang, I Hui Lee, Tzung Lieh Yeh, Yen Kuang Yang, Ru-Band Lu

**Affiliations:** 1 Department of Psychiatry, College of Medicine, National Cheng Kung University, Tainan, Taiwan; 2 Institute of Behavioral Medicine, National Cheng Kung University, Tainan, Taiwan; 3 Institute of Allied Health Sciences, College of Medicine, National Cheng Kung University, Tainan, Taiwan; 4 Addiction Research Center, College of Medicine, National Cheng Kung University, Tainan, Taiwan; 5 Department of Psychiatry, National Cheng Kung University Hospital, Tainan, Taiwan; 6 Department of Psychiatry, Tri-Service General Hospital, National Defense Medical Center, Taipei, Taiwan; 7 Department of Psychiatry, Tainan Hospital, Department of Health, Executive Yuan, Tainan, Taiwan

**Keywords:** Antisocial alcoholism, Serotonin, *Serotonin transporter gene*, *5-HT1B gene*

## Abstract

**Background:**

Several studies have hypothesized that genes regulating the components of the serotonin system, including *serotonin transporter* (*5-HTTLPR*) and *serotonin 1 B receptor (5-HT1B),* may be associated with alcoholism, but their results are contradictory because of alcoholism’s heterogeneity. Therefore, we examined whether the *5-HTTLPR* gene and *5-HT1B* gene *G861C* polymorphism are susceptibility factors for a specific subtype of alcoholism, antisocial alcoholism in Han Chinese in Taiwan.

**Methods:**

We recruited 273 Han Chinese male inmates with antisocial personality disorder (ASPD) [antisocial alcoholism (AS-ALC) group (*n* = 120) and antisocial non-alcoholism (AS-N-ALC) group (*n* = 153)] and 191 healthy male controls from the community. Genotyping was done using PCR-RFLP.

**Results:**

There were no significant differences in the genotypic frequency of the *5-HT1B G861C* polymorphism between the 3 groups. Although AS-ALC group members more frequently carried the *5-HTTLPR S/S, S/L*_*G*_*,* and *L*_*G*_*/L*_*G*_ genotypes than controls, the difference became non-significant after controlling for the covarying effects of age. However, the *5-HTTLPR S/S, S/L*_*G*_*,* and *L*_*G/*_*L*_*G*_ genotypes may have interacted with the *5-HT1B G861C C/C* polymorphism and increased the risk of becoming antisocial alcoholism.

**Conclusion:**

Our study suggests that neither the *5-HTTLPR* gene nor the *5-HT1B G861C* polymorphism alone is a risk factor for antisocial alcoholism in Taiwan’s Han Chinese population, but that the interaction between both genes may increase susceptibility to antisocial alcoholism.

## Background

Alcoholism (also: alcohol dependence) is a complex, heterogeneous disorder that is influenced by multiple genes as well as sociocultural factors [1,2]. Twin studies have shown that genetic factors account for about 50-60% of the overall variance in alcoholism [3-5]. Many candidate genes have been studied; however, the results are controversial. Therefore, to reduce the heterogeneity of alcoholism by proper subtyping is important. In addition, a gene-to-gene interaction approach may be more revealing than a single-gene approach for studying alcoholism. By using appropriate subtypes of alcoholism and the gene-to gene interaction approach, the results may be more conclusive.

Cloninger (1987) identified two subtypes of alcoholism. Type I alcoholism is characterized by late onset, more psychological dependence, and anxious/depressed personality traits. Individuals with type II alcoholism are characterized by early onset, a higher familial risk for alcoholism, and antisocial personality traits [1]. We previously subdivided alcoholism into three categories: pure, anxious/depressive, and antisocial (AS) [6]. We showed that antisocial alcoholism (AS-ALC; alcoholism comorbid with antisocial personality disorder), which overlaps to some extent with Cloninger’s type II alcoholism, have a strong genetic predisposition to alcoholism and different genetic polymorphisms than do other subtypes of alcoholism. The protective effect of the ADH1B*2 allele against alcoholism vanished in antisocial alcoholics [6], and the protective effect of the ALDH2*2 allele might disappear in individuals with antisocial personality disorder (ASPD) and carrying the MAOA-uVNTR 4-repeat allele [7]. Antisocial alcoholics had higher novelty-seeking scores with an interaction between the *DRD2 TaqI A1* (*A1A1* or *A1/A2*) and *5-HTTLPR* S/S genotype [8]. Moreover, antisocial alcoholics had different polymorphisms of the *serotonin (5-HT) receptor 1B A-161T* from other subtypes of alcoholics [9]. All the evidence supports the typology of alcoholism and the particular genetic vulnerability of antisocial alcoholics.

Numerous studies have reported reduced brain serotonin (5-hydroxytryptamine, 5-HT) function in patients who are similar to antisocial alcoholics. Lower cerebrospinal fluid concentration of the 5-HT metabolite 5-HIAA (5-hydroxyindoleacetic acid) has been found in early-onset antisocial alcoholics [10], impulsive alcoholic criminals [11,12], and alcoholic fire setters [13,14]. In addition, the variation of the central 5-HT system has been shown to modulate alcohol consumption and may contribute to the risk of alcoholism [15-17]. Genes encoding the 5-HTT gene (the serotonin transporter protein; genetic locus SLC6A4) and 5-HT receptor, which may cause different levels of activity of 5-HTT, have been examined as candidate genes for the risk of alcoholism. The polymorphism of the *5-HTT-linked promoter region (5-HTTLPR)*, which contains an insertion-deletion of two 22-bp repeats in a region of the promoter with multiple 22- to 23-bp repeats [18], affects the expression of serotonin transporter [19]. In vitro study, the short variant (S-allele) of the *5-HTTLPR* displayed reduced transcriptional activity [19] as well as decreased serotonin transporter expression and serotonin uptake in lymphoblast cell lines [18]. *5-HTTLPR* polymorphisms were also correlated to the function of serotonin transporter both in vivo imaging study [20] and in postmortem brain study [21]. Human SPECT study showed that homozygous carriers of the long allele (*L/L*) had an increase in the availability of raphe serotonin transporter [20]. Serotonin transporter mRNA levels in subjects with *S/L* and *S/S* genotypes were also lower than in subjects with the *L/L* genotype in postmortem brain study [21]. Although the S allele has been linked to a risk of alcoholism [22,23] and to antisocial alcoholics or type II alcoholics [24-26], this link is still controversial [20,27,28]. In addition to the long and short alleles, other subtypes of the L allele have been described [29]. The long variant with an adenosine at single nucleotide polymorphism (SNP) rs25531 (*L*_*A*_) has been reported to have higher activity than the long variant with a guanine at rs25531 (*L*_*G*_) [30,31]. Furthermore, other uncommon alleles that are longer than the L variant were found in several studies [29,32-35]. The extra-long allele or “novel allelic variant” were called ***XL*** variants with 15, 18,19, 20 or 22 repeats with unknown function [29,32-35]. Hence, the past dichotomous classification of *5HTTLPR* into *S* and *L* allele may not be sufficient and the association between the tri-allelic 5-HTTLPR polymorphism (*SL*_*A*_*L*_*G*_) and the risk of alcoholism or antisocial alcoholism remains unknown.

The serotonin 1B receptors (5-HT1B) are located on presynaptic and postsynaptic terminals and function as both autoreceptors and heteroreceptors that mediate the release of serotonin [36]. The *5-HT1B* gene has been associated with alcohol intake and aggression in animal and human studies. An alcohol preference locus has been mapped in mice to the region where *5-HT1B* is located [37]. *5-HT1B* receptor knockout mice were found to have increased aggressive behavior [38] and increased spontaneous alcohol consumption [39]. In human studies, of 16 reported polymorphisms of *5-HT1B*, the results of association studies that used the *G861C* polymorphism remain inconsistent [40]. Some studies associated polymorphisms of *5-HT1B G861C* with alcoholism [41] or antisocial alcoholism [42]. People with the *5-HT1B 861C* allele also had 20% fewer *5-HT1B* binding sites in the prefrontal cortex, which was the same as the alcoholism group [43,44]. However, subsequent studies did not confirm the association [45-47]. The heterogeneity of alcoholism, as well as different populations and ethnicities, may cause the discrepancy.

The prevalence rate of ASPD and antisocial behaviors in individuals with alcohol use disorders ranges from 20% to 33% [48-50]. Because of their high comorbidity, most studies have not distinguished the effects of ASPD from those of alcoholism in Western populations [51]. Being able to specify whether the genes were involved in pure ASPD (ASPD not comorbid with alcoholism (AS-N-ALC)) or AS-ALC, or both, may clarify the nature of these disorders and elucidate whether past genetic associations with AS-ALC were merely contributed by ASPD.

The aims of current study is to (1) investigate whether the tri-allelic *5-HTTLPR* polymorphism and *5-HT1B G861C* polymorphism are associated with AS-ALC in the Han Chinese population in Taiwan, and (2) assess the serotonergic genetic interaction between *5-HTTLPR* and *5-HT1B G861C* polymorphisms and antisocial alcoholism.

## Methods

### Participants and clinical assessments

This study was approved by the Institutional Review Boards for the Protection of Human Subjects at the Tri-Service General Hospital and National Cheng Kung University Hospital. Because of the low prevalence (0.1%) of ASPD in Han Chinese in Taiwan [52], it is difficult to recruit study participants with ASPD from clinics or from the community. Instead, we recruited 273 Han Chinese men with ASPD from a prison in Taipei (northern Taiwan) (*n* = 173) and a prison in Tainan (southern Taiwan) (*n* = 100). The research protocol was reviewed and approved by the Taiwan Ministry of Justice, which permitted us to collect blood samples from the participants. The procedures were fully explained to each participant before they gave written informed consent; however, there were no advocates for the inmates present when they were asked to sign the consent form. Participants were told that they were free to drop out of or to join the study at any time. We offered several compensatory benefits: 1) a free physical examination, 2) liver and renal function tests, and 3) and free treatment or assistance in transferring those with psychiatric or physical illnesses to hospitals. Each participant was initially evaluated by an attending psychiatrist and then interviewed by a psychologist well-trained in using the DSM-IV and the Modified Chinese Version of the Modified Schedule of Affective Disorder and Schizophrenia-Life Time (SADS-L) [53,54]. The inter-rater reliability of the Chinese version of the modified SADS-L was good with the kappa values range from 0.71 to 1.00 in several major mental illness [54]. The SADS-L was also found to be adequate for diagnosing ASPD with or without alcoholism [6,55-57]. Participants with major mental illnesses including schizophrenia, bipolar disorder, multiple substance abuse, or other organic mental disorders, and those who refused to participate (< 10%) were excluded from the study. None of the participants was taking any kind of medication. All who met the criteria for a diagnosis of antisocial personality disorder with or without alcoholism were assigned to the AS-ALC group) (*n* = 120) or the AS-N-ALC group (*n* = 153), respectively.

The healthy control group included 191 male volunteers recruited from the community. The Chinese version of the SADS-L was used to screen their psychiatric conditions. All controls were free of present and past major and minor mental illness (affective disorder, schizophrenia, anxiety disorder, personality disorder, alcohol abuse and dependence, and illegal substance use disorder), and none had a family history of psychiatric disorders among their first-degree relatives.

#### Blood samples and genotyping for 5-HTTLPR and 5-HT1B G861C polymorphisms

Twenty milliliters of blood was drawn from each participant, and standard methods were used to extract genomic DNA from the leukocytes. The genotypes of *5-HTTLPR* functional polymorphisms were genotyped using polymerase chain reaction plus restriction fragment length polymorphism (PCR-RFLP) analysis [18]. The A/G SNP of the *L* allele were determined using a modified protocol [58]. The *G861C* polymorphism of the *5-HT1B* gene was genotyped using a modified protocol [42].

### Statistical analyses

One-way analysis of variance (ANOVA) and the Bonferroni Post Hoc Test were used to determine the mean age differences between the subgroups. The difference in genotype frequency of *5-HTTLPR* and *5-HT1B G861C* polymorphisms between three groups was calculated using the Pearson χ^2^ test (2-tailed). The *5-HTTLPR* polymorphisms in AS-ALC and AS-N-ALC groups were further stratified by the *5-HT1B G861C* polymorphism using the χ^2^ tests. Logistic regression analysis was used to examine the main effect and gene-to-gene interaction of the *5-HTTLPR* and *5-HT1B G861C* polymorphisms for the risk of antisocial alcoholism. Both the *S*_*A*_ and the *S*_*G*_ alleles were recorded as the S allele because there is no functional difference in the SNPs [59]. Because past studies found that *S* and *L*_*G*_ carrier had lower function and expression of serotonin transporter [18-20,60], we divided the *5-HTTLPR* polymorphisms into 2 groups, low-functional (*S/SS/L*_*G*_*L*_*G*_*/L*_*G*_) and high-functional (*S/L*_*A*_*L*_*G*_*/L*_*A*_*L*_*A*_*/L*_*A*_*S/XLL*_*A*_*/XLL*_*G*_*/XL*), for data analysis. There was only 8 participants carried the *L*_*A*_*L*_*A*_ genotype in our study, so we did not separate the homozygous *L*_*A*_ carries from heterozygous *L*_*A*_ carriers into another group. SPSS for Windows 17.0 (SPSS Inc., Chicago, IL, USA) was used for all statistical analyses.

The power estimation was calculated using G-power 3.1 software [61,62]. Our total sample size (*n* = 464) had a power of 0.58 to detect a small effect (effect size = 0.1), of 0.99 to detect a medium effect (effect size = 0.3), and of 1.00 to detect a large effect (effect size = 0.5) of genotype distributions.

## Results

Healthy controls were significantly older than those in the AS-ALC (*P* = 0.004) and AS-N-ALC groups (*P* = 0.009; mean age: *F* = 6.87; *df* = 2, 461 (Table 1). A Bonferroni post hoc test did not show a significant age difference between the AS-ALC and AS-N-ALC groups.

**Table 1:** Demographic data

Group	*n*	Age (years)	*F*	*P*-value
AS-ALC	120	32.4 ± 7.6	6.87	0.001
AS-N-ALC	153	32.8 ± 7.5		
Controls	191	35.5 ± 9.4		

AS-ALC, antisocial alcoholism; AS-N-ALC, antisocial personality disorder without alcoholism.

Genotype distributions of *5-HTTLPR* and *5-HT1B G861C* were in Hardy-Weinberg equilibrium (*5-HTTLPR: P* = 0.75 in AS-ALC and *P* = 0.22 in AS-N-ALC; *5-HT1B G861C*: *P* = 0.07 in AS-ALC and *P* = 0.72 in AS-N-ALC). The distribution of the *5-HTTLPR* and *5-HT1B G861C* genotypes was not significantly different between the three groups (Table 2). However, there was a higher frequency of the low-functional *5-HTTLPR* polymorphism (*S/S*, *S/L*_*G*_, *L*_*G*_*/L*_*G*_) in the AS-ALC group than in the control group (Table 2). After stratifying the *5-HTTLPR* polymorphisms with the *5-HT1B G861C* polymorphism, a significant difference in the frequency of *5-HTTLPR* polymorphisms was found after controlling for the *5-HT1B G861CC/C* genotype for both AS-ALC versus controls and AS-ALC versus AS-N-ALC (Table 3). A logistic regression analysis, done to control the possible covarying effect of age, showed that the *5-HTTLPR* and *5-HT1B G861C* genotypes were not significantly different between the AS-ALC and control groups or the AS-ALC and the AS-N-ALC groups (Table 4). However, the regression did reveal a significant interaction between the *5-HTTLPR* and *5-HT1B G861C* genes. Compared with AS-N-ALC and control groups, the interaction between the *5-HTTLPR* low-functional group (*S/S*, *S/L*_*G*_, *L*_*G*_*/L*_*G*_ genotype) and the *5-HT1B G861C C/C* genotype may increase the risk of becoming an antisocial alcoholic (*P* = 0.04, odds ratio (OR) = 6.22 and 5.76, respectively) (Table 4).

**Table 2:** **Genotype distribution and frequency of the** ***5HTTLPR*** **and** ***5HT1B G861C*** **polymorphism**

Group	*n*	*5HTTLPR* genotype frequency (%)		χ²	*P*-value	*5HT1B G861C* genotype frequency (%)			χ²	*P*-value
		*S/S*, *S/L*_*G*_, *L*_*G*_*/L*_*G*_	*S/L*_*A*_, *L*_*G*_*/L*_*A*_, *L*_*A*_*/L*_*A*_, *S/XL*, *L*_*A*_*/XL*, *L*_*G*_*/XL*			*G/G*	*G/C*	*C/C*		
AS-ALC	120	90 (75.0)	30 (25.0)	5.39	0.02^a^*	25 (20.8)	70 (58.3)	25 (20.8)	4.58	0.10^a^
AS-N-ALC	153	100 (65.4)	53 (34.6)	2.95	0.09^b^	44 (28.8)	74 (48.4)	35 (22.9)	3.07	0.22^b^
Controls	191	119 (62.3)	72 (37.7)	5.50	0.06^c^	55 (28.8)	88 (46.1)	48 (25.1)	5.04	0.28^c^

AS-ALC: Antisocial alcoholism;

AS-N-ALC: Antisocial personality disorder without alcoholism.

^a^ AS-ALC vs. Healthy Controls.

^b^ AS-ALC vs. AS-N-ALC.

^c^ AS-ALC vs. AS-N-ALC vs. Controls.

*P < 0.05.

**Table 3:** ***5HTTLPR*** **genotype frequency after stratification of** ***5HT1B G861C*** **genotype**

*G861C* Genotype		*5HTTLPR* genotype (%)			
	Groups	*S/S*, *S/L*_*G*_, *L*_*G*_*/L*_*G*_	*S/L*_*A*_, *L*_*G*_*/L*_*A*_, *L*_*A*_*/L*_*A*_, *S/XL*, *L*_*A*_*/XL*, *L*_*G*_*/XL*	χ^2^	P
**AS-ALC vs. Controls**					
*G/G*	AS-ALC	15 (60.0)	10 (40.0)	0.023	0.878
	Controls	32 (58.2)	23 (41.8)		
*G/C*	AS-ALC	53 (75.7)	17 (24.3)	1.086	0.297
	Controls	60 (68.2)	28 (31.8)		
*C/C*	AS-ALC	22 (88.0)	3 (12.0)	7.509	0.006*
	Controls	27 (56.3)	21 (43.8)		
**AS-ALC vs. AS-N-ALC**					
*G/G*	AS-ALC	15 (60.0)	10 (40.0)	0.784	0.376
	AS-N-ALC	31 (70.5)	13 (29.5)		
*G/C*	AS-ALC	53 (75.7)	17 (24.3)	3.075	0.079
	AS-N-ALC	46 (62.2)	28 (37.8)		
*C/C*	AS-ALC	22 (88.0)	3 (12.0)	3.863	0.049*
	AS-N-ALC	23 (65.7)	12 (34.3)		

*P < 0.05

AS-ALC, antisocial alcoholism; AS-N-ALC, antisocial personality disorder without alcoholism.

**Table 4:** **Logistic regression analysis of** ***5HTTLPR*** **gene and** ***5HT1B*** **receptor gene** ***G861C*** **polymorphism and their interaction for the risk of antisocial alcoholism (AS-ALC)**

	AS-ALC			
**Models**	B	OR	95% CI	*P*-value
**Model 1**				
*5HTTLPR* low functional group	−0.47	0.62	0.22-1.75	0.37
*5HT1B G861C G/C*	−0.21	0.81	0.29-2.27	0.69
*5HT1B G861C C/C*	−1.13	0.32	0.07-1.46	0.14
*5HTTLPR* low-functional group*5HT1B G861C G/C	1.09	2.97	0.84-10.44	0.09
*5HTTLPR* low-functional group*5HT1B G861C C/C	1.83	6.22	1.10-35.27	0.04*
**Model 2**				
*5HTTLPR* low-functional group	0.14	1.15	0.43-3.07	0.78
*5HT1B G861C G/C*	0.49	1.63	0.61-4.35	0.33
*5HT1B G861C C/C*	−1.26	0.28	0.07-1.19	0.09
*5HTTLPR* low-functional group*5HT1B G861C G/C	0.07	1.07	0.32-3.65	0.91
*5HTTLPR* low-functional group*5HT1B G861C C/C	1.75	5.76	1.09-30.44	0.04*

*5HTTLPR* low-functional group: *S/S*, *S/L*_*G*_, *L*_*G*_*/L*_*G*_

*5HTTLPR* high-functional group: *S/L*_*A*_, *L*_*G*_*/L*_*A*_, *L*_*A*_*/L*_*A*_, *S/XL*, *L*_*A*_*/XL*, *L*_*G*_*/XL*

B, coefficients; CI, confidence interval; OR, odds ratio

Model 1, reference groups are: *5HTTLPR* high-functional group, *5HT1B G861C G/G* genotypes, and AS-N-ALC group; covarying for age

Model 2, reference groups are: *5HTTLPR* high-functional group, *5HT1B G861C G/G* genotypes, and controls; covarying for age

*P < 0.05.

## Discussion

We found that interaction between the *5-HTTLPRS/SS/L*_*G*_*L*_*G*_*/L*_*G*_ genotypes and the *5-HT1B G861C C/C* genotypes was associated with the AS-ALC group compared with the control and AS-N-ALC groups. Unlike past studies [24-26,43], we did not find an association between the *5-HTTLPR* or the *5-HT1B G861C* polymorphisms with AS-ALC. Although the AS-ALC group seemed to have a higher frequency of the *5-HTTLPRS/SS/L*_*G*_*L*_*G*_*/L*_*G*_ genotype than did the control group, when the effect of age was controlled by logistic regression, the difference was not significant.

The interaction between the *5-HTTLPRS/SS/L*_*G*_*L*_*G*_*/L*_*G*_ and the *5-HT1B G861C C/C* genotypes may result in reduced brain serotonin function. Because patients with the *5-HT1B 861 C* allele were found with fewer 5-HT1B binding sites in the prefrontal cortex [43,44] and the *5-HTTLPRS/SS/L*_*G*_*L*_*G*_*/L*_*G*_ was the low-functional group [30,31], our results imply that only the combined effect of serotonergic dysfunction from the studied genes, but not from individual genes, may be associated with risk for antisocial alcoholism. Several other reasons may also account for our positive finding. First, past studies compared the *5-HTTLPR* polymorphism between antisocial alcoholics and healthy (without ASPD and non-alcoholics) controls. Because ASPD has been linked to the *5-HTTLPR* polymorphism [63-65], we tried to control for the confounding effect of ASPD by comparing antisocial alcoholics with healthy controls and antisocial non-alcoholics. Second, our healthy control group belonged to a super-control group that excluded any other major or minor mental illness, while in other studies [25,26], the authors included only individuals from unscreened community controls and no antisocial non-alcoholics. Moreover, we used a tri-allelic functional *5-HTTLPR* polymorphism instead of a bi-allelic polymorphism. All these factors may have caused results different from those of other reports.

We found no association between antisocial alcoholism and the *5-HT1B G861C* polymorphism. A previous study [43] showed that the frequencies of the *5HT1B 861 C* allele varied widely among different ethnic groups, ranging from 20% in African-Americans to 36% in Hispanics and 42% in Asians. In our current study, the frequency of the *5-HT1B C* allele (48.9% in the control group, 49.6% in the AS-ALC group, and 47.1% in the AS-N-ALC group) was higher than in previous reports. We also added an AS-N-ALC group to decrease the effect of ASPD. Different populations and ethnicities may also partially explain our different results.

Our past study [66] showed that antisocial alcoholics and antisocial non-alcoholics shared a similar genetic vulnerability of dopamine-related genes, which was different from healthy controls. However, in the present study, we did not find the same distributions of the *5-HTTLPR* and *5-HT1B* genes in the three groups. It seems that the antisocial non-alcoholics and healthy controls had a similar genotype distribution of these serotonin-related genes, one different from that of the antisocial alcoholics. We may speculate that other serotonin-related genes have the same distribution between individuals with ASPD and the general population. In addition, serotonin may modulate the reinforcement processes, possibly through complex interactions with the mesolimbic dopamine system [60,67] and the regulatory functions of several serotonin receptor subtypes, including 5-HT1B receptor [68-70]. The interaction of the dopaminergic and serotonergic neurotransmitter systems in the nucleus accumbens and the ventral striatum is thought to be critical in addiction [60,70,71]. Furthermore, enzymes involved in dopamine metabolism will also metabolize serotonin. Serotonin is initially metabolized to 5-hydroxyindole-3-acetaldehyde (5-HIAL) by monoamine oxidase (MAO) [72], and then oxidized to 5-hydroxyindole-3-acetic acid (5-HIAA) by aldehyde dehydrogenase (ALDH) [73]. We have reported [74] that the *dopamine D2 receptor* gene interacted with the *5-HTTLPR* gene in antisocial alcoholics with specific personality traits. Hence, additional studies to explore the associations among the *5-HTTLPR*, the *5-HT1B* gene, 5-HT metabolic enzyme-related genes, and dopamine-related genes will be required to study the pathogenesis of AS-ALC.

Our study has several limitations. First, the study populations in the AS-ALC and AS-N-ALC groups were small. In addition, the cell size with the significant finding was also small, which may cause the risk of false positive results. Larger study populations are needed. Second, there were few participants with the *5-HTTLPRL*_*A*_*/L*_*A*_ genotype, and about 3.8% with the *5-HTTLPR XL* allele, which is different from other ethnic groups [30]. It may be feasible to divide those with the *5-HTTLPRS/L*_*A*_*L*_*G*_*/L*_*A*_*L*_*A*_*/L*_*A*_*S/XLL*_*A*_*/XLL*_*G*_*/XL* genotypes into a high-functional group and those with the *S/SS/L*_*G*_*L*_*G*_*/L*_*G*_ genotypes into a low-functional group for statistical analysis according to past study results [18-20,60]. Although the function of the *5-HTTLPR XL* allele remains unknown, one study had speculated that it may function as an *L* allele [75]. However, additional study will be required to test its function since a significant proportion of Han Chinese carry this allele [75]. Finally, it is very likely that individuals with ASPD may belong to a specific group since members of both the AS-ALC and AS-N-ALC groups were all recruited from jail, which caused sampling biases. In addition, we used controls without any past psychiatric history from the community. Regardless of alcoholism, the inmate participants with ASPD were substantially different from the healthy community participants [75]. It is necessary to be aware of the possibility of false-positive results when comparing these phenotypes with super-normal controls^42^.

Our results supported the notion that antisocial alcoholism is associated with the interaction between the *5-HTTLPR* and *5-HT1B G861* polymorphisms. Additional studies to examine the *5-HTTLPR* and *5HT1B G861C* gene polymorphisms and their interaction effects with the dopaminergic gene in antisocial alcoholism are required.

### Patient consent

Obtained.

### Ethical approval

This study was approved by the Institutional Review Boards for the Protection of Human Subjects at the Tri-Service General Hospital and National Cheng Kung University Hospital.

## Abbreviations

5-HTTLPR, Serotonin transporter; 5-HT1B, Serotonin 1 B receptor; ASPD, Antisocial personality disorder; AS-ALC, Antisocial alcoholism; AS-N-ALC, Antisocial non-alcoholism.

## Competing interests

The authors declare no conflict of interests.

## Authors’ contributions

The author, TYW wrote the first draft of this manuscript and designed this study with the author RBL. The authors, SYL and Y-HC managed the statistical analyses. The authors, SLC, SHC, CHC and CLC managed the lab work. The authors, TYW, SYH, NST, IHL, TLY, YKY and RBL managed the patients recruitment. All authors read and approved the final manuscript.
